# Specific IgA and metalloproteinase activity in bronchial secretions from stable chronic obstructive pulmonary disease patients colonized by *Haemophilus influenzae*

**DOI:** 10.1186/1465-9921-13-113

**Published:** 2012-12-11

**Authors:** Laura Millares, Alicia Marin, Judith Garcia-Aymerich, Jaume Sauleda, José Belda, Eduard Monsó

**Affiliations:** 1 Fundació Parc Taulí, Sabadell/Badalona, Spain; 2 CIBER de Enfermedades Respiratorias, CIBERES, Bunyola, Spain; 3 Universitat Autonoma de Barcelona, Esfera UAB, Barcelona, Spain; 4 Department of Respiratory Medicine, Hospital Universitari Germans Trias i Pujol, Badalona, Spain; 5 Centre for Research in Environmental Epidemiology (CREAL), Barcelona, Spain; 6 Municipal Institute of Medical Research (IMIM-Hospital del Mar), Barcelona, Spain; 7 Department of Experimental and Health Sciences, Universitat Pompeu Fabra, Barcelona, Spain; 8 CIBER Epidemiología y Salud Pública (CIBERESP), Barcelona, Spain; 9 Department of Respiratory Medicine, Hospital Universitari Son Espases, Palma de Mallorca, Spain; 10 Hospital Arnau de Vilanova, Valencia, Spain; 11 Department of Respiratory Medicine, Hospital Universitari Parc Taulí, Sabadell, Spain

**Keywords:** Chronic Obstructive Pulmonary Disease (COPD), *Haemophilus influenzae*, Secretory IgA, Metalloproteinase-9 (MMP-9), Tissue-inhibitor of metalloproteinases-1 (TIMP-1)

## Abstract

**Background:**

*Haemophilus influenzae* is the most common colonizing bacteria of the bronchial tree in chronic obstructive pulmonary disease (COPD), and positive cultures for this potentially pathogenic microorganism (PPM) has been associated with local inflammation changes that may influence the relationships between *H. influenzae* and the bronchial mucosa.

**Methods:**

A cross-sectional analysis of stable COPD patients enrolled in the Phenotype and Course of Chronic Obstructive Pulmonary Disease (PAC-COPD) Study, focusing on bronchial colonization by *H. influenzae,* was performed. Specific IgA against the PPM was measured by optical density, and metalloproteinase-9 (MMP-9) and tissue inhibitor of metalloproteinase-1 (TIMP-1) using ELISA in sputum samples. Levels in patients colonized by *H. influenzae* and non-colonized patients were compared.

**Results:**

Sputum supernatant for the measurement of specific IgA against *H. influenzae* was available from 54 stable COPD patients, who showed levels of specific IgA significantly lower in colonized (n=21) than in non-colonized patients (n=33) (15 [4-37] versus 31 [10-75], p=0.033, Mann-Whitney U test). Proenzyme MMP-9 was measured in 44 patients, and it was higher in colonized (n=12, 1903 [1488-6699] ng/ml) than in non-colonized patients (n=32, 639 [373-972] ng/ml) (p<0.001, Mann-Whitney U test). Active form of MMP-9 was also higher in colonized (126 [25-277] ng/ml) than in non-colonized patients (39 [14-68] ng/ml) (p=0.021, Mann-Whitney U test), and the molar ratio between proenzyme MMP-9 and TIMP-1 was above 1 (2.1 [0.1-12.5]) in colonized patients, significantly higher than the ratio found in non-colonized patients (0.2 [0.08-0.5]) (p=0.030, Mann-Whitney U test).

**Conclusions:**

Clinically stable COPD patients colonized by *H. influenzae* had lower levels of specific IgA against the microorganism and higher values of the active form of MMP-9 in their sputum supernatant than non-colonized patients. Bronchial colonization by *H. influenzae* may cause structural changes in the extracellular matrix through a defective defense and the production of active metalloproteinases.

## Background

Potential pathogenic microorganisms (PPMs) colonize the bronchial tree of COPD patients and are found in the bronchial secretions of one third of adults with stable COPD, a rate that increases with the worsening of airflow obstruction [1]. *H. influenzae* is the most common colonizing bacteria isolated from these patients, and is also frequently recovered when exacerbation symptoms appear [2]. This PPM is able to adapt to changing environments through gene expression changes [3-5], some of which modify its virulence [6,7].

Both microorganism and host factors determine the outcome of the acquisition of a *H. influenzae* strain by the bronchial tree [8]. The bronchial mucosa is protected by a specialized immune system focused on the prevention of colonization and infection by PPMs, being antibodies the first line of this defense. IgA is the principal immunoglobulin produced in the bronchial tissue and a key element in this mechanism [9,10], with a major role in host defenses through inhibition of microbial adherence, toxin inactivation and promotion of humoral immunity [11]. The protection of bronchial mucosa from *H. influenzae* is partly mediated by immune exclusion [12], an essentially mechanical process in which secretory IgA (sIgA) agglutinates bacteria allowing the entrapment of the created bacterial complexes in mucus, which are expelled through mucociliary clearance. Under certain conditions *H. influenzae* may produce specific enzymes that cleave human IgA1, a subclass of bronchial IgA, separating the antigen recognition fragments of the immunoglobulin from its constant region and inactivating its protective role [13-15]. This direct effect of the proteases produced by *H. influenzae* on the levels of IgA may be clinically significant in the pathogenesis of COPD in colonized and infected patients.

The presence of *H. influenzae* in the bronchial tree of stable COPD patients is associated with an inflammatory response [16]. In colonized patients an imbalance between endogenous proteinases and proteinase inhibitors may be found that interferes with normal tissue function and repair [17]. Matrix metalloproteinases (MMPs) are a family of Ca^2+^-activated, Zn^2+^-dependent proteases which are secreted by a wide variety of cells and are capable of degrading all components of the extracellular matrix [18]. Their activity is physiologically controlled by tissue inhibitors of metalloproteinases (TIMPs), but in pathological conditions a switch in MMP production and activity may occur, which may lead to abnormal tissue destruction [19]. MMPs are thought to participate in the excessive collagenolytic and elastolytic activity found in COPD, as suggested by the high levels in lung tissue and induced sputum of patients with this disease [20-22]. Among the MMP family, MMP-9 is responsible for tissue repair and remodeling through the degradation of basement membrane type IV collagen and other matrix proteins. TIMP-1 is the major endogenous inhibitor of both MMP-8 and MMP-9, and high levels of this protein have been found in COPD [23].

With the hypothesis that in stable COPD bronchial colonization by *H. influenzae* may be related to an impaired local specific immunoglobulin response and to an imbalance between MMP-9 and TIM-1 levels in bronchial secretions, we carried out a cross-sectional analysis of specific IgA against *H. influenzae* and metalloproteinase activity in sputum samples recovered from patients included in the PAC-COPD Study. Specific IgA and concentrations of the MMP-9, both total and active, and its inhibitor TIMP-1 were measured in sputum supernatant recovered from stable COPD patients colonized and non-colonized by *H. influenzae.* The PAC-COPD Study comprises patients who had a first admission for COPD exacerbation and who were examined later after the stabilization of the disease.

## Methods

### Design and participants

This cross-sectional analysis of the relationships between bronchial colonization by *H. influenzae* in COPD, local production of specific IgA against this PPM and metalloproteinase activity is part of the population-based Phenotype and Course of Chronic Obstructive Pulmonary Disease (PAC-COPD) Study. The PAC-COPD Study focus on patients who are in a moderate stage of their disease and had not required repeated admissions when examined, and with this purpose enrolled 342 COPD patients hospitalized for the first time for an exacerbation of their disease in nine teaching hospitals in Spain, who were evaluated later when they were clinically stable. The recruitment process and the definitions of COPD, exacerbation and first admission in the PAC-COPD Study have been reported elsewhere [24,25]. Stable patients from the PAC-COPD Study who expectorated samples with low squamous cell content and had complete information on sputum microbiology and inflammatory mediators at the baseline evaluation were identified [26] and selected for the analysis when they presented bronchial colonization by *H. influenzae* or negative sputum cultures. Bronchial colonization by other PPMs showed high heterogeneity and low prevalence figures (<10%) in the PAC-COPD Study, and were excluded from the analysis. The research protocol was approved by the ethics committees of all participating hospitals and written informed consent was obtained from all subjects.

### Clinical and functional variables

Patients were enrolled at their first hospital admission for COPD exacerbation and answered during this admission an epidemiologic questionnaire that covered smoking habits, respiratory symptoms and treatments the previous year, performing all clinical tests at least three months after hospital discharge and when clinically stable. Functional characteristics assessed included results of forced spirometry and reversibility testing, which were performed before sputum induction. Detailed information about the sources of the questionnaires and standardization of the tests used in the PAC-COPD study has been published elsewhere [24,27].

### Sputum induction

Spontaneous sputum was collected for microbiology and for measurements of specific immunity against *H. influenzae* and metalloproteinase activity. In patients unable to produce sputum spontaneously a sample was induced according to standard methods [28-30]. Patients were pre-treated with an inhaled ß-adrenergic agent 10 minutes before nebulization of increasing concentrations of saline (0.9%, 3%, 4% and 5%), for seven minutes each in order to induce sputum. Patients were asked to blow their nose, rinse their mouth, and swallow water before the procedure, and the nebulization was interrupted when the sputum volume collected was 1 ml or more [31]. After each induction the patient attempted to cough up sputum into a sterile plastic dish. The first sputum sample was taken for the microbiologic exam and later sputum samples were used to analyze inflammatory markers, specific immunity and metalloproteinases. Recovered sputum was processed within 60 minutes of collection to guarantee cell viability [32], that was determined by trypan blue exclusion in a Neubauer hemocytometer [24,25], and kept at 4°C when stored. Under the assumption that samples with 20% squamous cells or fewer were representative of tracheobronchial secretions [33], stable patients who expectorated samples with squamous cell contents below that limit were selected for the present analysis.

### Microbiology and inflammatory markers in sputum

Sputum samples were weighed, processed with an equal volume of dithiothreitol (DTT) (Sputasol, Oxoid Ltd., Hants, UK), and cultured according to standard methods. Microbiological processing included determination of microbial typology and load through serial dilutions and culture in selective media for PPMs, according to standard methods [34] with quantitative cultures expressed as colony-forming units (cfu) per milliliter. Cultures were considered positive for bronchial colonization by *H.influenzae* when loads were 100 cfu/ml or higher.

Sputum was separated from contaminating saliva by macroscopic examination. Selected sputum was mixed with four times its weight of DTT solution and vortexed. A weight of phosphate-buffered saline solution equal to that of DTT was then added, and the whole mixture was further vortexed. The suspension was filtered through a 48-μm nylon gauze. Total inflammatory cell count, expressed as the absolute number of cells per gram of sputum, was calculated by subtracting squamous cells from the total cell count. Absolute and differential cell counts for neutrophils were calculated by counting 400 non-squamous cells on Wright-stained slides. The remaining suspension was centrifuged at 750*g* and the supernatant was decanted and stored at –80°C.

Cytokine concentrations (interleukin [IL] -1β, IL-6, IL-8) were measured in the supernatant using a cytokine bead array (BD Biosciences, San Diego CA, USA). The detection limits of these assays were 7.2 pg/ml for IL-1β, 2.5 pg/ml for IL-6 and 3.6 pg/ml for IL-8. All assays were performed in duplicate and reported values correspond to the average of the two determinations.

### Specific IgA against *Haemophilus influenzae* in sputum

**Figure 1:**
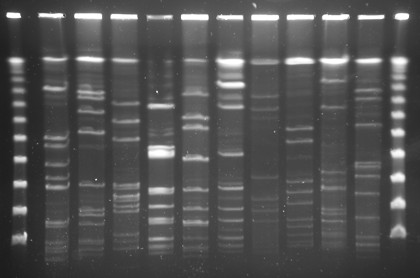
**Ten distinct isolates of *H. Influenzae*, isolated from COPD patients, used as capture antigen in ELISA testing.**

ELISA was used to determine the IgA antibody level against *H. influenzae* in the supernatant of the recovered sputum samples. The capture antigen was prepared as follows: ten distinct isolates of *H. influenzae*, distinguished by different pulsed-field gel electrophoresis patterns (Figure 1), were grown overnight on chocolate agar plates at 37°C and 5% CO_2_. Several colonies were inoculated to brain heart infusion broth supplemented with 10 μg/ml of hemin and β-nicotinamide adenine dinucleotide (NAD) (Sigma-Aldrich, Saint Louis, Missouri) and incubated at 37°C. Bacterial concentration was adjusted to optical density (OD) _~_0.1 at a wavelength of 625 nm (_~_10^8^ cfu/ml) and the ten isolates were mixed to create a pooled sample of *H. influenzae* strains. Fifty μl of this suspension were used to coat each well on a microtiter plate (Nunc Brand, Denmark) and the plate was incubated overnight at 37°C. Between each step the wells were washed three times with PBS (400μl/well) and in all following steps the reagents used were added to the wells in volumes of 100 μl. The wells were blocked by addition of 200 μl of PBS-1% bovine serum albumin (BSA) and incubated at 37°C for two hours. The wells were then washed, and sputum supernatants diluted in PBS were added and incubated for 1 h at 37°C. After washing, goat-anti human IgA (Sigma) diluted 1:1000 in PBS-1%BSA was added and incubated 1 h at 37°C. Following another washing, anti-goat conjugated to alkaline phosphatase (Sigma) diluted 1:5000 in PBS-1%BSA was added and incubated 1 h at 37°C. Color was developed after washing by adding a developing buffer containing p-Nitrophenyl phosphate (Sigma), and optical density (OD) read at 405 nm. All samples were run in triplicate and the final result was the average of the three scores. To control for day-to-day variability, an isolate of *H. influenzae* and its homologous supernatant was tested again in each plate and used as an internal control, accepting variabilities below 15% [35].

Specific IgA determinations required the availability of a volume equal or over 25 microliter of supernatant, and were performed in samples from the studied patients and in a sputum supernatant pool of nine healthy controls from general population who were used as the normal reference. Results were expressed as the OD ratio between patients and the healthy control reference (OD patients-OD pool of healthy controls/OD pool of healthy controls).

### Metalloproteinase activity in sputum

Concentrations of MMP-9, both of its proenzyme of 92 kDA (proMMP-9) and its active form of 68 kDa, and of their inhibitor TIMP-1, were determined in the sputum supernatant using a commercially available enzyme-linked immunosorbent assay (ELISA) kit (Amersham Biosciences, GE Healthcare, Buckinghamshire, UK), and carried out according to the manufacture´s recommendations. These determinations required a volume equal or over 100 microliter of supernatant, and had detection limits of 0.5 ng/ml for MMP-9 and 3.13 ng/ml for TIMP-1.

ProMMP-9 to TIMP-1 molar ratios below 1:1 keep MMP-9 below its activity level and are the reference in healthy subjects [36]. Ratios above 1:1 are considered abnormal and are associated with availability of the active form of MMP-9 in bronchial secretions [37].

### Statistical analysis

Data were analyzed using the SPSS statistical software package version 18 (SPSS Inc., Chicago, IL, USA). Results for categorical variables are expressed as absolute and relative frequencies, and results for continuous variables as means and standard deviations (SD), or as medians and percentiles 25-75 (P25-P75) when the distribution was not normal.

First, clinical and functional variables and bronchial inflammation markers of patients showing colonization by *H. influenzae* and non-colonized patients were compared, and the difference in the specific IgA response against *H. influenzae* in sputum found in patients colonized and non-colonized by this PPM was assessed. The ratio between the level of specific IgA against *H. influenzae* in patients and the level in healthy subjects (OD patients-OD pool of healthy controls/OD pool of healthy controls) was calculated in both groups and used for the comparison. Finally, the concentrations of proMMP-9, TIMP-1 and active MMP-9 in patients colonized by *H. influenzae* were compared with the levels found in non-colonized patients. The molar ratio between MMP-9 and TIMP-1 was additionally calculated and also used for the comparisons between patients colonized by these PPMs and non-colonized patients. All analyses were performed using chi-square, Fisher exact or Mann-Whitney U tests as required. Statistical tests were two-sided, and a p value of 0.05 or less was reported as statistically significant.

## Results

### Sputum quality

One hundred thirty-three participants in the PAC-COPD Study produced good quality sputum samples which showed a low proportion of squamous cells (2 [0.5-6]) and high cell viability (79 [64-90]), which were considered as representative of bronchial secretions. They were COPD patients with an average age of 70 (9) years and moderate lung function impairment (mean post-bronchodilator forced expiratory volume in one second [FEV_1_] 52% [SD 16] of predicted) (Table 1). In 39 of these patients, PPMs were recovered from sputum (29.3%), and in 22 (17%) of them *H. influenzae* was the bacterium recovered.

**Table 1:** Clinical, functional and inflammatory characteristics of bronchial secretions in patients with representative sputum samples* (n=133)

**Patient characteristics**	
Age (years), mean (SD)	70 (9)
Males, n (%)	124 (93)
Smoking pack-years, median (P25-P75)	67 (43-102)
Current smoker, n (%)	35 (27)
Exacerbation last year ≥1, n (%)	45 (34)
FEV_1_ post-BD % pred, mean (SD)	52 (16)
**Sputum characteristics**	
Neutrophils/ml ×10^6^, median (P25-P75)	6.4 (1.7-17.1)
Neutrophils %, median (P25-P75)	72 (48-84)
IL-1β (pg/ml), median (P25-P75)	209 (52-695)
IL-6 (pg/ml), median (P25-P75)	124 (54-259)
IL-8 (×10^3^) (pg/ml), median (P25-P75)	11 (4-16)
Colonized by PPMs, n (%)	39 (29.3)
Colonized by *H. influenzae*, n (%)	22 (17)
Bacterial load of *H. influenzae* x10^6^ (cfu/ml), median (P25-P75) ^†*^	5.4 (1-12)

*Patients with representative sputum samples according to percentage of squamous cells≤20%.

†* In patients with a positive culture for *H. influenzae*. Expressed as ×10^6^ cfu/ml.

Definition of abbreviations: SD = standard deviation; P25-P75 = percentile 25-percentile 75; BD = bronchodilator; FEV_1_= forced expiratory volume in 1 second; IL = interleukin; PPMs = potential pathogenic microorganisms; cfu = colony forming units.

**Figure 2:**
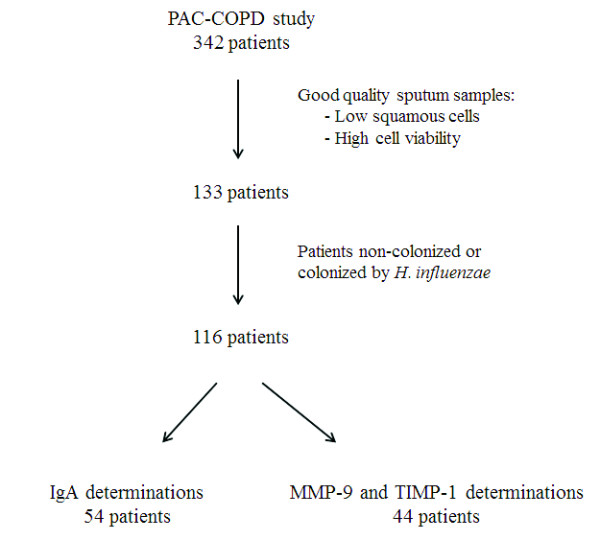
Flow chart showing the number of subjects used in each analysis.

Patients with bronchial colonization by *H. influenzae* or negative sputum cultures were the target population for the analysis of specific IgA (n=116), and sputum supernatant for the measurement of specific IgA against *H. influenzae* was available from 54 of these patients (46.5%), 21 colonized by the PPM and 33 non-colonized, who were the population subsample analyzed. In 44 patients sputum availability allowed also the determination of MMP-9 and TIMP-1 (38%), which were measured in 12 patients colonized by *H. influenzae* and in 32 non-colonized patients (Figure 2). The comparisons of smoking habits (pack-year), respiratory symptoms (exacerbations in the year previous to admission), and forced spirometry (postbronchodilator FEV_1_%) in patients with specific IgA measured, in patients who had metalloproteinase activity determined, and in patients without sputum availability for the present analyses (n=62) did not show statistically significant differences (data not shown).

### Clinical characteristics and bronchial inflammation according to colonization

Clinical differences between patients colonized by *H. influenzae* and non-colonized patients were not significant, except for higher cumulative smoking in colonized patients (103 [60-138] vs. 58 [36-90] pack-years; p= 0.002) (Table 2). A similar well-defined neutrophilic inflammatory pattern in sputum was observed in both colonized and non-colonized patients, with significantly higher median values for IL-1β (746 [121-1802] vs. 154 [41-485] pg/ml; p=0.001) and IL-8 (16 [11-22] vs. 8 [3-15] (x10^3^) pg/ml; p< 0.001) in patients colonized by *H. influenzae*.

**Table 2:** **Patient characteristics and inflammatory mediators in sputum according to bronchial colonization by *Haemophilus influenzae *(n=116)**

	**Non-colonized**	**Colonized by *H. influenzae***	p value*
	**n=94**	**n=22**	**-**
Patient characteristics			
Age (years), mean (SD)	69 (9)	70 (8)	0.880
Current smoker, n (%)	24 (26)	6 (27)	0.932
Smoking pack-years, median (P25-P75)	58 (36-90)	103 (60-138)	0.002
FEV_1_ post-BD % pred, mean (SD)	52 (16)	50 (15)	0.597
Inhaled corticosteroids, n (%)	11 (12)	2 (9)	0.727
Inflammatory mediators in sputum			
Total cells/ml × 10^6^, median (P25-P75)	12 (4-24)	7 (4-22)	0.388
Neutrophils/ml ×10^6^, median (P25-P75)	7 (2-18)	4 (1-14)	0.526
IL-1β (pg/ml), median (P25-P75)	154 (41-485)	746 (121-1802)	<0.001
IL-6 (pg/ml), median (P25-P75)	109 (50-201)	168 (53-436)	0.172
IL-8 (×10^3^) (pg/ml), median (P25-P75)	8 (3-15)	16 (11-22)	< 0.001

*Analyses performed using chi-square, Fisher exact or Mann-Whitney U tests as required.

### Specific IgA against *H. influenzae* in bronchial secretions

**Figure 3:**
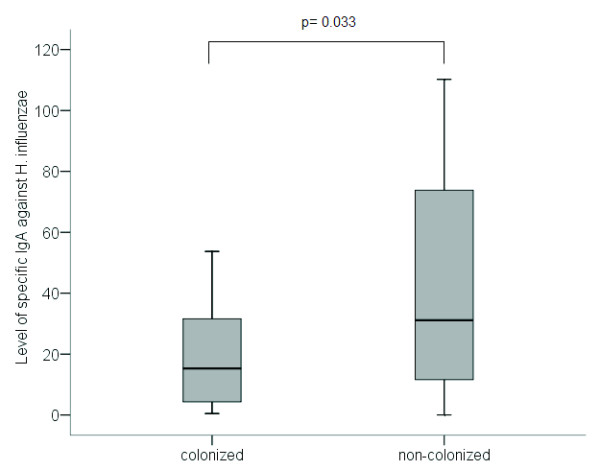
**Level of specific IgA against *H. influenzae *in patients colonized by this bacteria (n=21) and non-colonized patients (n=33).** Results expressed as optical density (OD) ratio between patients and healthy controls.

Level of IgA antibody against whole-cell *H. influenzae* antigens in the sputum of colonized and non-colonized patients was related to the level of IgA against this PPM in the sputum samples of nine healthy controls. IgA levels were significantly lower in patients colonized by *H. influenzae* (15 [4-37]) than in non-colonized patients (31 [10-75]) (p=0.033, Mann-Whitney U test) (Figure 3).

### Sputum metalloproteinase activity

**Figure 4:**
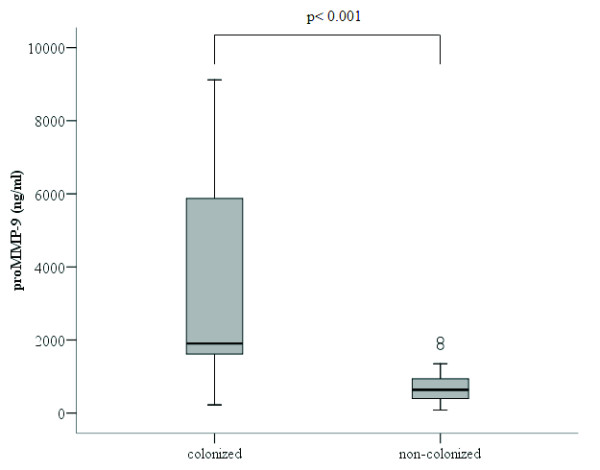
**Proenzyme MMP-9 concentration in the sputum supernatant of patients colonized by *H. influenzae *(n=12) and non-colonized patients (n=32).**

**Figure 5:**
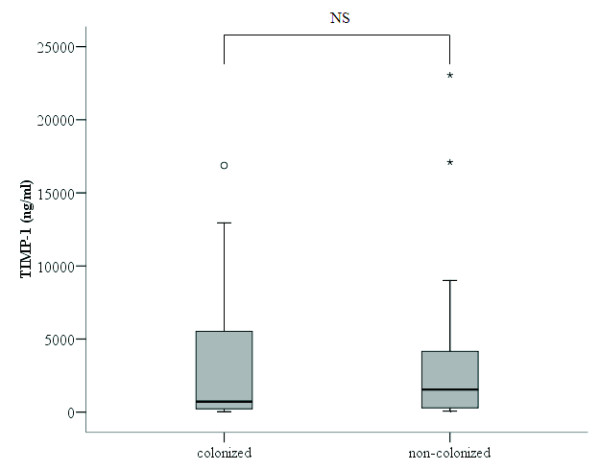
**TIMP-1 concentration in the sputum supernatant of patients colonized by *H. influenzae *(n=12) and non-colonized patients (n=32).**

**Figure 6:**
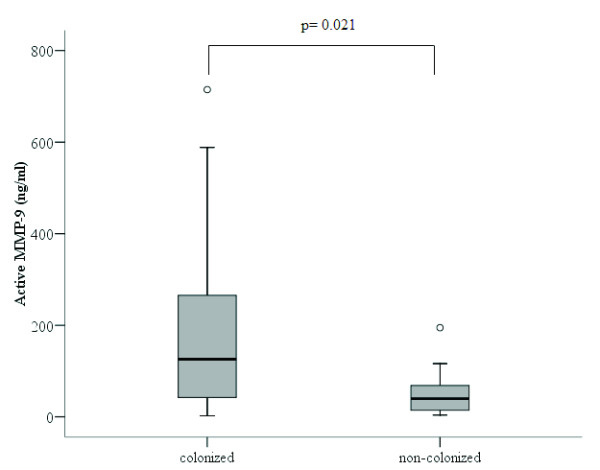
**Active MMP-9 concentration in the sputum supernatant of patients colonized by *H. influenzae *(n=12) and non- colonized patients (n=32).**

The concentration of proenzyme MMP-9 was significantly higher in patients colonized by *H. influenzae* (1903 [1488-6699] ng/ml) than in non-colonized patients (639 [373-972] ng/ml) (p<0.001, Mann-Whitney U test) (Figure 4). No differences were found in the levels of TIMP-1 between these groups (Figure 5). When the level of the active form of MMP-9 was assessed, it was also higher in patients colonized by *H. influenzae* (126 [25-277] ng/ml) than in non-colonized patients (39 [14-68] ng/ml), a comparison that reached statistical significance (p=0.021, Mann-Whitney U test) (Figure 6).

**Figure 7:**
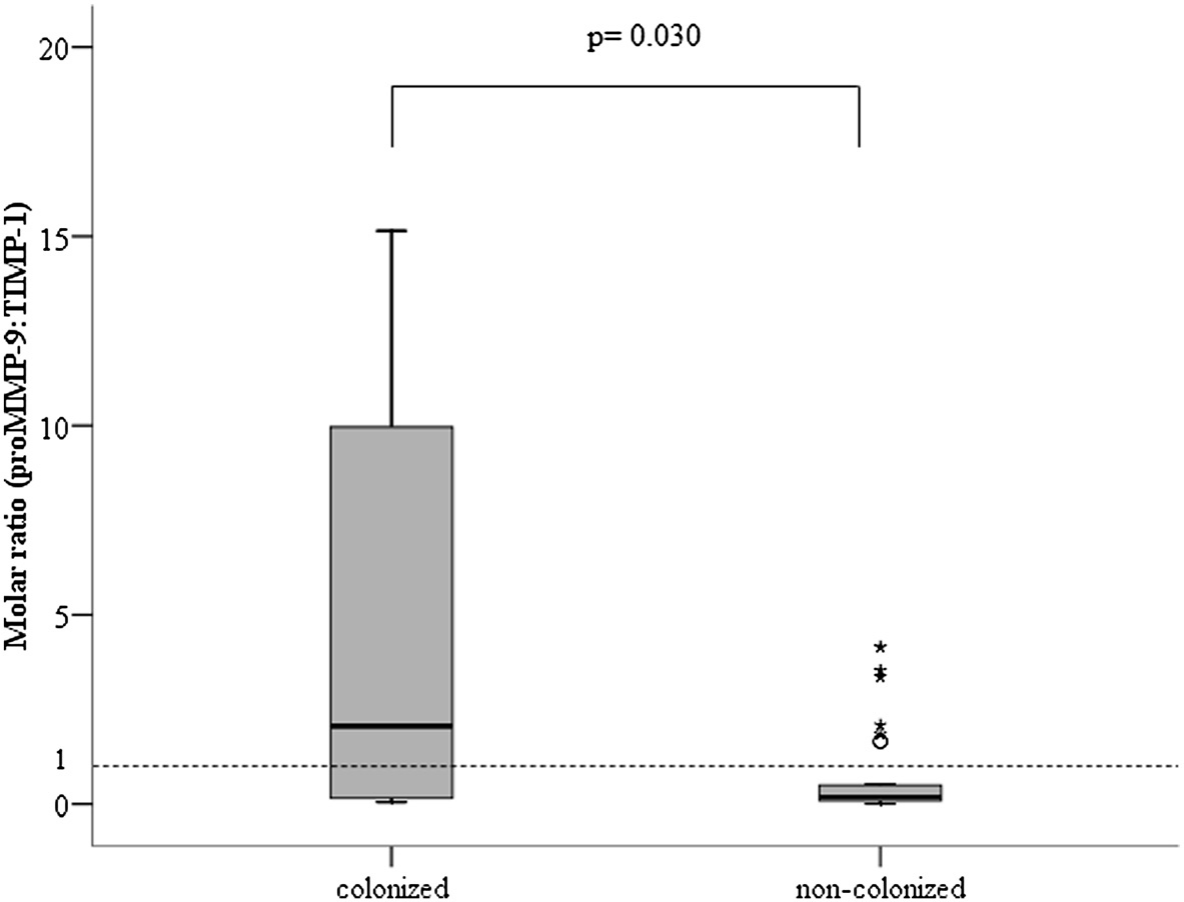
**Molar ratio between proenzyme MMP-9 and TIMP-1 in colonized patients colonized by *H. influenzae *(n=12) and non-colonized patients (n=32).**

The calculation of the molar ratio between proMMP-9 and TIMP-1 showed that bronchial colonization by *H. influenzae* was associated with pro MMP-9/TIMP-1 ratios above 1:1 (2.1 [0.1-12.5]), which were significantly higher than the ratios found in non-colonized patients (0.2 [0.08-0.5]) (p=0.030, Mann-Whitney U test) (Figure 7).

## Discussion

Sputum samples of a well-characterized cohort of stable COPD patients who has not required repeated admissions for exacerbation of their disease has been analyzed in the present study. Relationships between bronchial colonization by *H. influenzae*, the specific IgA response against this PPM, and metalloproteinase activity in bronchial secretions were examined. Bronchial colonization by *H. influenzae* was associated with low levels of IgA against this PPM in sputum, a defective specific immunologic response that may difficult the eradication of *H. influenzae* from the bronchial tree. Levels of proMMP-9 and its active form, additionally, were high in patients colonized by *H. influenzae*, supporting a significant impact of this PPM on the metalloproteinase activity in bronchial secretions, in the absence of exacerbation symptoms. These findings suggest that bronchial colonization by *H. influenzae* may be related to a defective immune response against this PPM, and associated with an increase in MMP-9 levels to concentrations that may cause extracellular matrix destruction and airway remodeling.

The low levels of specific IgA against *H. influenzae* found in the present study in colonized patients may be related to protease production by the PPM or due to a defective response of the bronchial mucosa. The immune response of the bronchial tree is the first defense line for the protection of the respiratory system, with a major role of local immunoglobulins, functionally related to the epithelial barrier and mucociliary clearance [38]. Secretory IgA originates from the selective transport of polymeric IgA across the epithelial cells, and is the predominant type of antibody in the bronchial mucosa. This immunoglobulin agglutinates colonizing bacteria and participates in the inhibition of bacterial mucosal adherence [34]. Strains of *H. influenzae* produce IgA proteases [39,40], and levels of sIgA in bronchial secretions are partly influenced by the production of specific enzymes that cleave the subtype 1 of this immunoglobulin (IgA1), separating the antigen recognition fragments from the constant region of IgA, in patients colonized by this PPM [10,14,15,41-43]. Because the first line of the specific immunologic defense against *H. influenzae* is provided by sIgA [9], determinants of the level of this immunoglobulin in bronchial secretions, related to protease production, local consumption or defective production may play a role in the pathogenesis of bronchial colonization and infection in COPD [44], an hypothesis supported by the results of the present study.

We have found high levels of the remodeling protein proMMP-9 and its active form in stable COPD patients colonized by *H. influenzae*. Previous studies have reported a relationship between bronchial colonization and inflammation in these patients, identifiable through high levels of IL-1β and IL-8 in bronchial secretions [45-47], with a higher effect when *H. influenzae* is the colonizer [26]. Interleukin 1β, among other mediators, stimulate the release of MMP from alveolar macrophages, and upregulate MMP activity in the COPD airway [18]. Destruction of small bronchi and alveoli, leading to emphysema, involves members of the MMP family [36], and there is significant evidence that these proteases play a significant role in COPD pathogenesis. Transgenic mice over-expressing MMP-1 develop emphysema [48], whilst MMP-12 knockout mice are protected from emphysema despite prolonged cigarette smoke exposure [45]. High levels of MMP-8, -9 and TIMP-1 have been found in COPD [17,49], and MMP-9 level in bronchial secretions was higher in smokers with COPD than in smokers without functional limitation [50,51]. A marked increase in MMP-9 expression and activity in lung parenchyma and increased MMP-9/TIMP-1 ratios in induced sputum have been also reported in patients with COPD, when compared with healthy subjects [19]. Interaction with TIMPs is the physiological way to control the proteolytic activity of MMPs in normal conditions, and an imbalance between MMPs and TIMPs has been proposed as the cause of the increased levels of MMP-9 that are often detected in COPD patients [23,37,49,52,53]. TIMP-1 is the major endogenous inhibitor of MMP-8 and MMP-9, and the levels of this protein are usually elevated in COPD [23]. As far as we know, the present study is the first to examine the relationship between colonization by *H. influenzae*, MMP-9 and TIMP-1 in bronchial secretions of stable COPD patients. We have found high levels of both proMMP-9 and its active form in patients colonized by *H. influenzae*, suggesting that the presence of *H. influenzae* in the airways of stable COPD patients may have an effect on tissue matrix. The molar ratio between proMMP-9 and TIMP-1 was also found higher in patients colonized by *H. influenzae* in the present study. ProMMP-9 to TIMP-1 ratios below 1:1 keep the metalloproteinase below its activity level and are the reference in healthy subjects [36]. Accordingly, values above 1:1 must be considered abnormal and associated with availability of the active form of MMP-9 in bronchial secretions, at levels that may be high enough to be associated with disease [37].

Limitations of the performed study should be considered. Firstly, the present study is cross-sectional, and the correlation between low levels of IgA and persistent or recurrent colonization by *H. influenzae* cannot be concluded from the available data. Secondly, the PAC-COPD cohort included COPD patients who had mainly moderate disease and has been hospitalized because an exacerbation only once, and this characteristic determines that the obtained results may not be applicable to patients with advanced COPD, who also show a higher prevalence of bronchial colonization due to other PPMs*,* which are unusual in patients with moderate disease. The proportion of females in the PAC-COPD Study is low, a common finding in epidemiologic studies performed in Spain, attributable to the delayed introduction of smoking habits in women in the region, and determines that the observed results may not be extrapolated to female COPD patients. Finally, we have only analyzed the IgA response against *H. Influenzae*, because other COPD colonizers had low figures in the PAC-COPD Study. Therefore, further studies will be necessary to analyze the specific immunity against other PPMs in COPD patients.

## Conclusions

In conclusion, our clinically stable COPD patients colonized by *H. influenzae* had lower levels of specific IgA against this microorganism and higher values of the active form of MMP-9 in the sputum supernatant than non-colonized patients. *H. influenzae* colonization may be facilitated by this defective specific immune response, and the colonization by this PPM may cause structural changes in the extracellular matrix through the stimulation of metalloproteinase activity.

## Competing interests

The authors declare that they have no competing interests.

## Authors’ contributions

LM carried out the experimental assays, performed the statistical analysis and drafted the manuscript. AM contributed to the patient selection and management, interpretation of data and database handling. JGA participated in the conception and design of PAC-COPD study and was involved in database handling, statistical analysis and quality control. JS and JB were involved in conception and design of the methodology of the study. EM designed and coordinated the study and helped to draft the manuscript. All authors revised the manuscript critically for important intellectual content, and read and approved the final manuscript.

## Authors’ information

The Phenotype and Course of COPD (PAC-COPD) Study Group: Centre for Research in Environmental Epidemiology (CREAL), Barcelona: Josep M Antó (Principal Investigator), Judith Garcia-Aymerich (project coordinator), Marta Benet, Jordi de Batlle, Ignasi Serra, David Donaire-Gonzalez, Stefano Guerra; Hospital del Mar-IMIM, Barcelona: Joaquim Gea (center coordinator), Eva Balcells, Angel Gayete, Mauricio Orozco-Levi, Ivan Vollmer; Hospital Clínic-Institut D’Investigacions Biomèdiques August Pi i Sunyer (IDIBAPS), Barcelona: Joan Albert Barberà (center coordinator), Federico P Gómez, Carles Paré, Josep Roca, Robert Rodriguez-Roisin, Xavier Freixa, Diego A Rodriguez, Elena Gimeno, Karina Portillo; Hospital General Universitari Vall D’Hebron, Barcelona: Jaume Ferrer (center coordinator), Jordi Andreu, Esther Pallissa, Esther Rodríguez; Hospital de la Santa Creu i Sant Pau, Barcelona: Pere Casan (center coordinator), Rosa Güell, Ana Giménez; Hospital Universitari Germans Trias i Pujol, Badalona: Eduard Monsó (center coordinator), Alicia Marín, Sara Barea, Josep Morera; Hospital Universitari de Bellvitge, L’Hospitalet de Llobregat: Eva Farrero (center coordinator), Joan Escarrabill; Hospital de Sabadell, Corporació Parc Taulí, Institut Universitari Parc Taulí (Universitat Autònoma de Barcelona), Sabadell: Antoni Ferrer (center coordinator); Hospital Universitari Son Dureta, Palma de Mallorca: Jaume Sauleda (center coordinator), Àlvar G Agustí, Bernat Togores; Hospital de Cruces, Barakaldo: Juan Bautista Gáldiz (center coordinator), Lorena López; Hospital General Universitari, València: José Belda
